# New dual peroxisome proliferator activated receptor agonist—Saroglitazar in diabetic dyslipidemia and non-alcoholic fatty liver disease: integrated analysis of the real world evidence

**DOI:** 10.1186/s12933-019-0884-3

**Published:** 2019-06-17

**Authors:** Upendra Kaul, Deven Parmar, K. Manjunath, Mitesh Shah, Krupi Parmar, Kishor P. Patil, Ashok Jaiswal

**Affiliations:** 1 Batra Hospital and Medical Research Centre, New Delhi, India; 2 Zydus Discovery DMCC, Dubai, UAE; 3 Zydus Research Centre, Cadila Healthcare Limited, Ahmedabad, India; 4 Zydus Healthcare Limited, Zydus Tower, CTS No-460/6 of Village Pahadi, Off I. B. Patel Road, Goregaon (East), Mumbai, 400 063 India

**Keywords:** Saroglitazar, Dual PPAR agonist, Diabetic dyslipidemia, Triglyceride, Glycosylated hemoglobin, Alanine aminotransferase, Non-alcoholic fatty liver disease

## Abstract

**Background:**

Saroglitazar, a novel dual peroxisome proliferator activated receptor (PPAR) agonist, in clinical trials, has shown an improvement in lipid and glycemic parameters through the PPAR-α and γ agonist actions, respectively. It was granted marketing authorization in India in 2013 for diabetic dyslipidemia. This review was conducted to summarize the effects of Saroglitazar in patients with diabetic dyslipidemia in real world clinical studies conducted after marketing authorization in India.

**Methods:**

In this review, we selected real world clinical studies of Saroglitazar published as manuscripts and abstracts presented at scientific conferences. In all these studies, patients with diabetic dyslipidemia were treated with Saroglitazar 4 mg once daily for at least 12 weeks and different lipid and glycemic parameters were measured at the baseline and end of the study.

**Results:**

In 18 selected studies (5 published manuscripts and 13 abstracts), a total of 5824 patients with diabetic dyslipidemia were prescribed Saroglitazar 4 mg for a duration ranging from 12 to 58 weeks. Across all the studies, mean age of patients ranged from 49.6 to 59.1 years and the proportion of female patients ranged from 22% to 42%. Across all the studies, there was a consistent mean reduction in triglyceride levels (~ 45% to 62%), total cholesterol levels (~ 17% to 26%), non-high-density lipoprotein cholesterol levels (~ 21% to 36%), low-density lipoprotein cholesterol levels (~ 11% to 27%), and glycosylated hemoglobin levels (~ 0.7% to 1.6%) with an increase in mean high-density lipoprotein cholesterol levels (up to 9%) from baseline to end of the study. Saroglitazar also improved alanine aminotransferase levels and fatty liver (evaluated by FibroScan™) in non-alcoholic fatty liver disease patients with diabetic dyslipidemia. Body weight remained unchanged and no significant adverse events (AEs) were reported in the studies.

**Conclusion:**

Saroglitazar effectively improved lipid and glycemic parameters without significant AEs in patients with diabetic dyslipidemia in real-world clinical studies of up to 58 weeks duration.

## Background

Cardiovascular diseases (CVDs) have emerged as the leading cause of morbidity and mortality worldwide with 80% of the disease burden occurring in low and middle-income countries [1]. Type 2 diabetes mellitus (T2DM) and dyslipidemia are now established risk factors of CVDs [1, 2]. With the rising prevalence of T2DM and dyslipidemia, CVDs have emerged as major public health threats worldwide [3–5]. Dyslipidemia in T2DM, also known as diabetic dyslipidemia, is characterised by high levels of triglyceride (TG) and small-dense low-density lipoprotein cholesterol (sd-LDL-C), low levels of high-density lipoprotein cholesterol (HDL-C), and increased insulin resistance, all of which increase the risk of CVDs [6]. Despite the success of statins in achieving the guideline recommended LDL-C goals, patients with dyslipidemia remain at a high residual risk of developing CVDs and this risk is further increased in T2DM patients [7–9]. Therefore, treatments targeting high TG, high non-HDL-C, and low HDL-C could be more effective in reducing the residual risk for future CVDs [7–9].

In recent years, dual peroxisome proliferator activated receptor (PPAR) α/γ agonists have attracted global attention as promising new treatment options for diabetic dyslipidemia due to a unique mechanism of action in improving lipid and glucose profile simultaneously [9, 10]. PPAR-α agonist action improves lipid profile, whereas PPAR-γ agonist action improves glucose profile in patients with diabetic dyslipidemia [9, 10]. Many dual PPAR α/γ agonists were developed but failed during preclinical stage or the clinical development stage due to lack of efficacy or safety issues [9, 10]. Saroglitazar, developed by Zydus Cadila, is a novel dual PPAR α/γ agonist (predominant PPAR-α and moderate PPAR-γ actions), aiming to improve lipid and glucose profiles without significant weight gain and edema (common in PPAR-γ agonists such as thiazolidinediones) [9].

Preclinical studies and Phase-1 & Phase-2 clinical trials demonstrated favourable effects of Saroglitazar on lipid and glycemic parameters [9, 11, 12]. In a Phase-3 clinical trial (PRESS V) in patients with diabetic dyslipidemia, Saroglitazar 2 mg and 4 mg significantly reduced TG from baseline to week-24 by 26% and 45%, respectively [11]. In another Phase-3 clinical trial (PRESS VI) in patients with diabetic dyslipidemia not controlled with Atorvastatin 10 mg, Saroglitazar 2 mg and 4 mg significantly reduced TG from baseline to week-12 by 45%. [12]. In both Phase-3 clinical trials, Saroglitazar also improved other lipid parameters and glucose parameters [9, 11, 12].

Following successful clinical trials, Saroglitazar was granted marketing authorization in India in 2013 and is indicated for the management of diabetic dyslipidemia and hypertriglyceridemia in T2DM not controlled by statin alone [9]. Since 2013, clinicians/clinical researchers have studied the effects of Saroglitazar in real world clinical settings in India. This review was done to summarize the effects of Saroglitazar in patients with diabetic dyslipidemia in real world clinical studies conducted in India after marketing authorization.

## Methods

### Study selection criteria

The studies included were observational studies based on real world clinical settings conducted after marketing approval of Saroglitazar in India. These studies were published as manuscripts or abstracts presented at the scientific conferences. In all these studies, patients with diabetic dyslipidemia were treated with Saroglitazar 4 mg once daily for at least 12 weeks and different lipid and glycemic parameters were measured at the baseline and end of the study. A few studies where adult patients with diabetic dyslipidemia were also diagnosed with non-alcoholic fatty liver disease (NAFLD) have also been included. Excluded studies were those that could not be categorised as real world clinical setting studies, such as bioanalytical studies, preclinical studies, randomized controlled trials (RCTs), review articles, and editorials.

### Search methods

We conducted a literature search for Saroglitazar studies in the PubMed in the National Center for Biotechnology Information (NCBI) databases (https://www.ncbi.nlm.nih.gov/) using the terms ‘Saroglitazar AND Observational Study’. Moreover, we also conducted a manual search for Saroglitazar studies in other electronic search engines (example, google scholar, google). In addition to published articles of Saroglitazar, we also conducted a manual search for abstracts of Saroglitazar studies presented at the global annual scientific congress.

### Study selection, data extraction, and analysis

#### Selection of studies

Two independent reviewers conducted the study eligibility assessment.

#### Data extraction

Two independent reviewers extracted data on relevant population characteristics, treatments, and outcomes from the studies. Data were extracted for the following variables: total number of patients; study duration; age; sex; body weight; patients on statins, patients on anti-diabetics, TG and other lipid parameters (LDL-C, HDL-C, total cholesterol (TC), non-HDL-C), glycemic parameter (glycosylated hemoglobin [HbA1c]), and liver parameter (alanine aminotransferase [ALT]). Data on adverse events (AEs) were also extracted. Each reviewer performed the quality check of data extracted by the other reviewer.

#### Statistical analysis

Demographics and baseline characteristics were presented as mean ± standard deviation (or mean) or number (percentage). Graphical representation was prepared for following variables: TG, non-HDL-C, TC, HDL-C, LDL-C, HbA1c, ALT, and body weight. For better graphical representation, all the included studies were grouped according to study duration (Table 1). Baseline mean value and end of the study assessment mean value were plotted for these study groups in the graphs (example, for 36 weeks study, baseline mean TG value and mean TG value at week-36 were plotted in the graph). The weighted mean was estimated for groups having more than one study in any of the groups as mentioned in Table 1.

**Table 1:** Selected real world clinical studies of Saroglitazar

Group	Study duration	Number of studies	Selected studies
Group 1	12 weeks	4	Shetty et al. [13], Thacker et al. [14], Joshi et al. [15], Bhattacharyya et al. [16]
Group 2	24 weeks	6	Saboo et al. [17], Joshi et al. [18], Chhaya et al. [19], Mohit et al. [20], Kaul et al. [21], Goyal et al. [22]
Group 3	27 weeks	1	Chatterjee et al. [23]
Group 4	36 weeks	1	Joshi et al. [24]
Group 5	40 weeks	1	Chatterjee et al. [25]
Group 6	52 weeks	4	Joshi et al. [26], Aneja et al. [27], Maheshwari et al. [28], Chatterjee et al. [29]
Group 7	58 weeks	1	Chatterjee et al. [30]

## Results

### Selection of studies

**Fig. 1:**
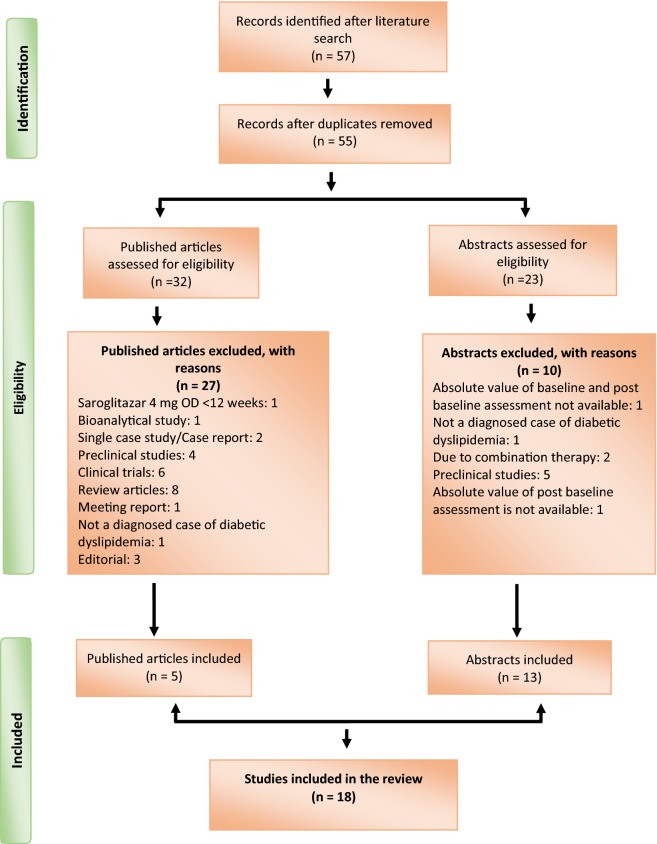
Study selection flowchart

We identified a total of 57 articles after initial literature search (Fig. 1). Among 57 articles, 18 articles were included in this review after the verification of the study eligibility criteria: 5 published full text articles and 13 abstracts. Figure 1 shows the flowchart of selecting eligible articles.

### Study population

Table 2 shows demographics and baseline characteristics from all the selected studies. A total of 5824 adult patients were prescribed Saroglitazar 4 mg once daily for durations ranging from 12 to 58 weeks across all the selected studies. Mean age of patients ranged from 49.6 to 59.1 years and the proportion of female patients ranged from 22 to 42% across all the studies. At baseline, patients on anti-diabetics ranged from 89 to 100% and on statins ranged from 39 to 100% across the studies.

**Table 2:** Demographics and baseline characteristics

Author (year)	N	D (W)	Age^a^ (years)	Female^b^ (%)	Weight^a^ (Kg)	Patients on statin^b^ (%)	TG^a^ (mg/dL)	LDL-C^a^ (mg/dL)	HDL-C^a^ (mg/dL)	TC^a^ (mg/dL)	Non-HDL-C^a^ (mg/dL)	HbA1c^a^ (%)	ALT^a^ (IU/L)
Shetty et al. (2015) [13]	2804	12	53.0 ± 10.0	37.5	72.3 ± 11.5	57.8	312.3 ± 122.7	139.5 ± 42.2	38.8 ± 8.7	240.2 ± 63.0	201.8 ± 64.1	8.3 ± 1.3	
Thacker et al. (2016) [14]	50	12	49.6	42.0		100.0	272.0	88.8	39.3	159.9		7.5	68.8
Joshi et al. (2018) [15]	18	12					1265.9 ± 394.3				320.8 ± 172.8	8.9 ± 1.7	
Bhattacharyya et al. (2018) [16]	60	12			79.1 ± 9.6		219.9 ± 178.8	108.3 ± 46.9	38.9 ± 9.8	175.9 ± 56.9	157.3 ± 53.4	7.9 ± 1.5	
Saboo et al. (2015) [17]	31	24					259.3 ± 37.9					9.0 ± 1.3	64.1 ± 6.2
Joshi et al. (2016) [18]	221	24	58.0	41.6		48.0	321.0					8.9	89.0
Chhaya et al. (2017) [19]	78	24			78.6 ± 9.8	85.0	348.7 ± 162.7	103.4 ± 26.0		192.8 ± 61.9		8.7 ± 1.4	
Mohit et al. (2017) [20]	50	24	58.6 ± 14.0	42.0	74.2 ± 13.6	100.0	212.9 ± 47.6	157.1 ± 75.6	48.9 ± 12.7	224.6 ± 28.1		8.8 ± 0.5	
Kaul et al. (2019) [21]	104	24	59.1 ± 11.4	22.1	73.2 ± 11.2	100.0	357.0 ± 332.0	91.0 ± 37.0	37.5 ± 16.6	176.0 ± 62.0	140.0 ± 55.0	7.9 ± 1.6	
Goyal et al. (2019) [22]	84	24	51.4 ± 10.3	26.2			334.7 ± 74.0					7.9 ± 0.5	98.0 ± 32.0
Chatterjee et al. (2015) [23]	31	27	54.0 ± 9.9	42.0	69.4 ± 9.9	68.0	335.5 ± 161.2	111.4 ± 46.9	39.2 ± 10.7	197.5 ± 52.6	161.4 ± 52.8	8.1 ± 1.8	52.1 ± 26.7
Joshi et al. (2015) [24]	787	36	53.0	35.6	73.9 ± 11.9	50.2	297.9 ± 122.6	132.5 ± 47.9	41.0 ± 14.7	239.9 ± 74.7	199.0 ± 76.5	8.5 ± 1.4	
Chatterjee et al. (2016) [25]	74	40	52.4 ± 9.6	37.8	68.0 ± 10.5	65.0	343.3 ± 211.7	105.2 ± 41.9	38.3 ± 10.6	186.3 ± 50.8	150.2 ± 50.4	7.8 ± 1.7	43.2 ± 24.2
Joshi et al. (2015) [26]	236	52	52.0 ± 10.0			38.6	316.0 ± 139.0	131.8 ± 43.8	42.5 ± 8.7		194.0 ± 48.5	8.5 ± 1.1	
Aneja et al. (2016) [27]	81	52	55.4	28.4		81.5	294.1 ± 83.9		41.4 ± 4.6	224.9 ± 29.9	183.1 ± 33.8	8.1 ± 0.7	
Maheshwari et al. (2016) [28]	106	52	54.8	34.0		99.0	252.7 ± 58.3	165.7 ± 49.7		223.8 ± 33.9	164.4 ± 33.9	8.2	
Chatterjee et al. (2017) [29]	851	52	53.0		66.8		295.1 ± 101.9	140.1 ± 31.4		234.6 ± 41.9	199.3 ± 42.5	11.3 ± 2.2	
Chatterjee et al. (2018) [30]	158	58	51.3 ± 10.9	32.3	70.5 ± 2.1	74.7	315.4 ± 176.3	101.8 ± 41.9	38.4 ± 10.2	180.7 ± 50.9	139.5 ± 56.3	7.9 ± 1.6	42.2 ± 26.6

Blank cells indicate data not available in the respective studies

N: Total number of patients on Saroglitazar 4 mg; D: study duration; W: weeks; M: mean; SD: standard deviation; TG: triglyceride; TC: total cholesterol; LDL-C: low-density lipoprotein cholesterol; HDL-C: high-density lipoprotein cholesterol; non-HDL-C: non high-density lipoprotein cholesterol; HbA1c: glycosylated hemoglobin; ALT: alanine aminotransferase

^a^Data for age, weight, TG, LDL-C, HDL-C, TC, non-HDL-C, HbA1c and ALT are presented in M or M ± SD

^b^Data for female and patients on statin are presented in percentage (%)

### Lipid parameters

**Fig. 2:**
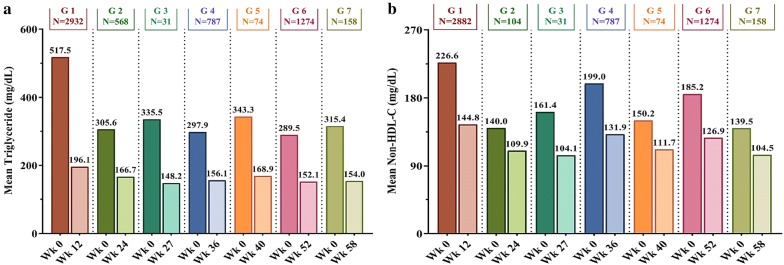
Mean change from baseline in triglyceride (**a**) and non-HDL-C (**b**). Non-HDL-C: Non-high-density lipoprotein cholesterol. Studies included [13–30]: Group 1 (12 weeks): Shetty et al. [13], Thacker et al. [14], Joshi et al. [15], Bhattacharyya et al. [16]; Group 2 (24 weeks): Saboo et al. [17], Joshi et al. [18], Chhaya et al. [19], Mohit et al. [20], Kaul et al. [21], Goyal et al. [22]; Group 3 (27 weeks): Chatterjee et al. [23]; Group 4 (36 weeks): Joshi et al. [24]; Group 5 (40 weeks): Chatterjee et al. [25]; Group 6 (52 weeks): Joshi et al. [26], Aneja et al. [27], Maheshwari et al. [28], Chatterjee et al. [29]; Group 7 (58 weeks): Chatterjee et al. [30]. Mean non-HDL-C calculation: data not available for Thacker et al. [14], Saboo et al. [17], Joshi et al. [18], Chhaya et al. [19], Mohit et al. [20], Goyal et al. [22]

**Fig. 3:**
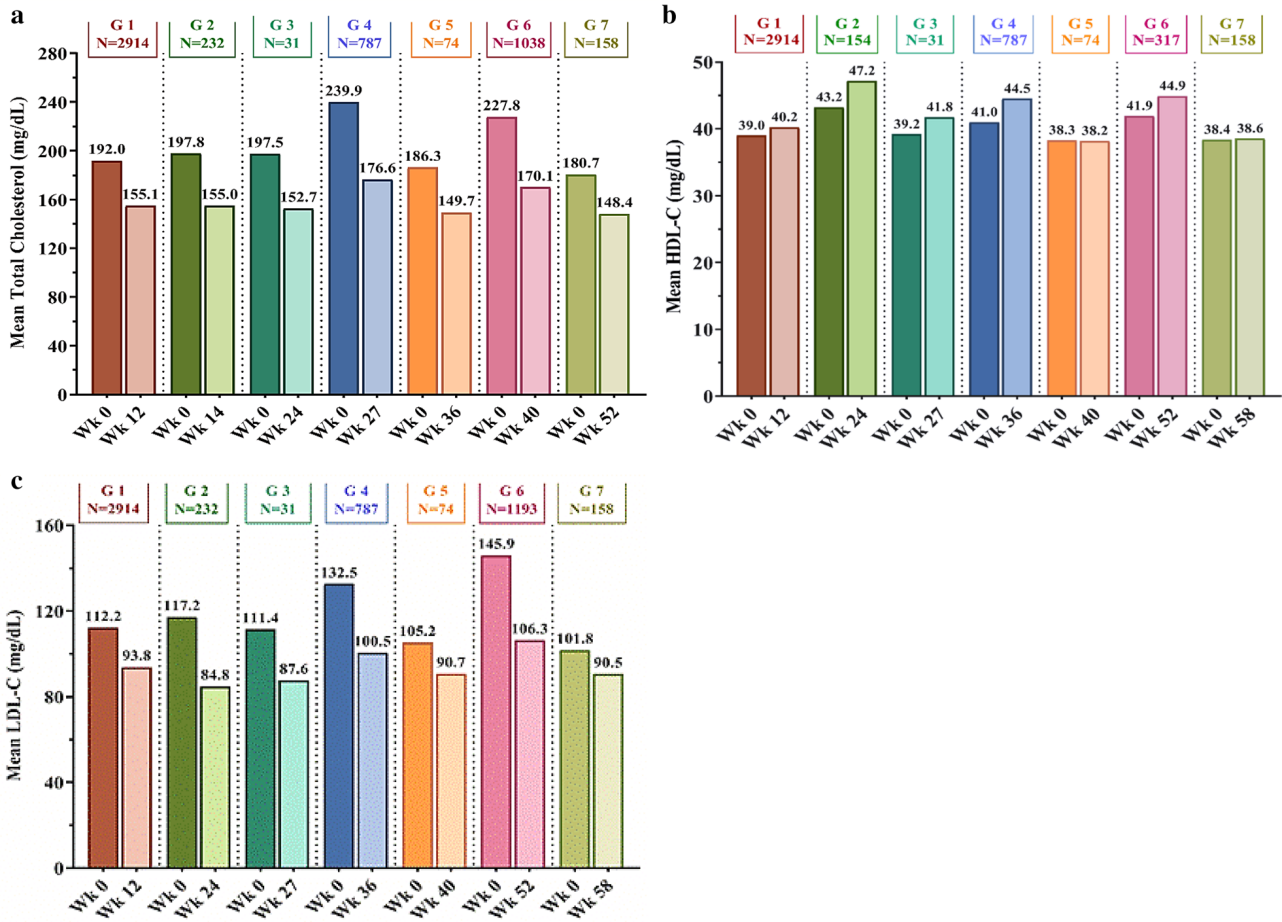
Mean change from baseline in total cholesterol (**a**), HDL-C (**b**), and LDL-C (**c**). HDL-C: High-density lipoprotein cholesterol; LDL-C: low-density lipoprotein cholesterol. Studies included [13–30]: Group 1 (12 weeks): Shetty et al. [13], Thacker et al. [14], Joshi et al. [15], Bhattacharyya et al. [16]; Group 2 (24 weeks): Saboo et al. [17], Joshi et al. [18], Chhaya et al. [19], Mohit et al. [20], Kaul et al. [21], Goyal et al. [22]; Group 3 (27 weeks): Chatterjee et al. [23]; Group 4 (36 weeks): Joshi et al. [24]; Group 5 (40 weeks): Chatterjee et al. [25]; Group 6 (52 weeks): Joshi et al. [26], Aneja et al. [27], Maheshwari et al. [28], Chatterjee et al. [29]; Group 7 (58 weeks): Chatterjee et al. [30]. Mean total cholesterol calculation: data not available for Joshi et al. [15], Saboo et al. [17], Joshi et al. [18], Goyal et al. [22], Joshi et al. [24]. Mean HDL-C calculation: data not available for Joshi et al. [15], Saboo et al. [17], Joshi et al. [18], Chhaya et al. [19], Goyal et al. [22], Maheshwari et al. [28], Chatterjee et al. [29]. Mean LDL-C calculation: data not available for Joshi et al. [15], Saboo et al. [17], Joshi et al. [18], Goyal et al. [22], Aneja et al. [27]

Across all the selected studies, there was a consistent mean reduction in TG levels (~ 45% to 62%), non-HDL-C levels (~ 21% to 36%), TC levels (~ 17% to 26%), LDL-C levels (~ 11% to 27%) with an increase in mean HDL-C levels (up to 9%) from baseline to week 12–58 (end of the study of the respective study groups) (Figs. 2 and 3).

### Glycemic parameter

**Fig. 4:**
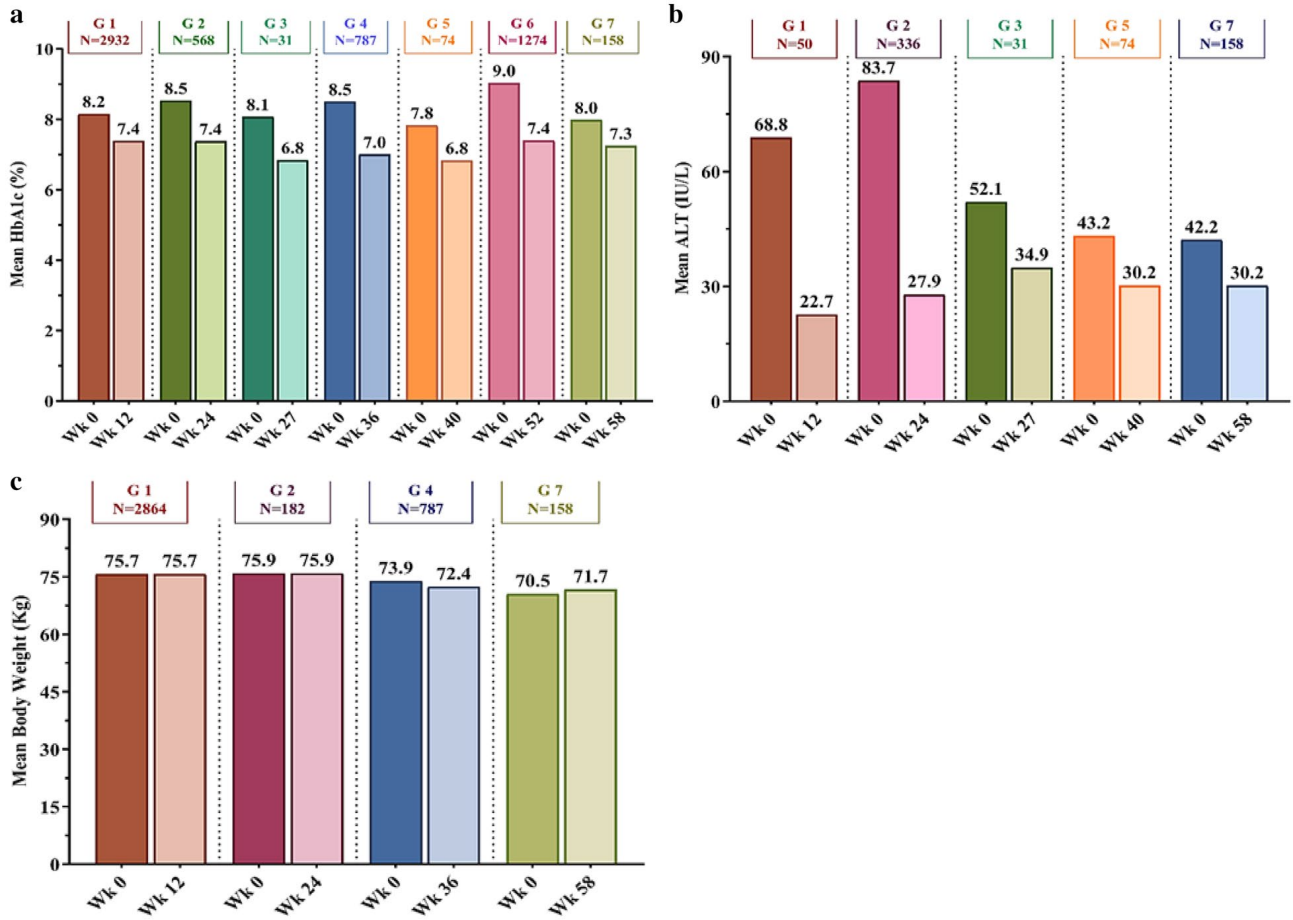
Mean change from baseline in HbA1c (**a**), ALT (**b**), and body weight (**c**). HbA1c: Glycosylated hemoglobin; ALT: alanine aminotransferase. Studies included [13–30]: Group 1 (12 weeks): Shetty et al. [13], Thacker et al. [14], Joshi et al. [15], Bhattacharyya et al. [16]; Group 2 (24 weeks): Saboo et al. [17], Joshi et al. [18], Chhaya et al. [19], Mohit et al. [20], Kaul et al. [21], Goyal et al. [22]; Group 3 (27 weeks): Chatterjee et al. [23]; Group 4 (36 weeks): Joshi et al. [24]; Group 5 (40 weeks): Chatterjee et al. [25]; Group 6 (52 weeks): Joshi et al. [26], Aneja et al. [27], Maheshwari et al. [28], Chatterjee et al. [29]; Group 7 (58 weeks): Chatterjee et al. [30]. Mean ALT calculation: data available for Thacker et al. [14], Saboo et al. [17], Joshi et al. [18], Goyal et al. [22], Chatterjee et al. [23]; Chatterjee et al. [25]; Chatterjee et al. [30]. Mean body weight calculation: data available for Shetty et al. [13], Bhattacharyya et al. [16], Chhaya et al. [19], Mohit et al. [20], Kaul et al. [21], Chatterjee et al. [23], Joshi et al. [24], Chatterjee et al. [25], Chatterjee et al. [29], Chatterjee et al. [30]

Across all the selected studies, there was a consistent mean reduction in HbA1c levels (~ 0.7% to 1.6%) from baseline to week 12–58 (end of the study of the respective study groups) (Fig. 4).

### Liver parameter

Across all the selected studies, there was a consistent mean reduction in ALT levels (~ 28% to 67%) from baseline to week 12–58 (end of the study of the respective study groups) (Fig. 4).

### Safety profile

Across all the selected studies, there was no change in mean body weight from baseline to week 12–58 (end of the study of the respective study groups) (Fig. 4). Chhaya et al. [19] reported knee joint pain in 2 patients. Kaul et al. [21] reported occasional chest discomfort/chest pain in 2 patients, burning sole in 1 patient, hypoglycemia after first dosing in 1 patient, and occasional suffocation in 1 patient. Kaul et al. [21] found hypoglycemia after first dosing to be Saroglitazar related AE.

## Discussion

Fibrates have been prescribed as lipid modifying agents for more than four decades; however, they have low potency and limited selectivity for PPAR-α [31–33]. Moreover, Fibrates have been associated with increased risk of myopathy, cholelithiasis, venous thrombosis, decline in renal function, and elevated transaminase concentrations [33]. Pemafibrate, a new novel selective PPAR-α modulator with high potency, could emerge as a more effective and safe alternative to Fibrates for management of dyslipidemia [34].

Thiazolidinediones, structural analogs of Fibrates, reduce insulin resistance and improve blood glucose levels through PPAR-γ agonist action [31–33]. However, thiazolidinediones have been associated with weight gain and peripheral edema [32, 33]. Moreover, Rosiglitazone was also associated with increased risk of myocardial infarction and has been withdrawn from Europe [33]. There has been a global concern over Pioglitazone use due to possibility of increased risk of heart failure and bladder cancer [33, 35].

Clinical evidences from Bezafibrate (a pan—PPAR [α, β/δ, γ] activator) studies supported the concept of a pan-PPAR/dual-PPAR therapeutic approach for diabetic dyslipidemia [31, 32]. In the past two decades, Glitazars, dual PPAR α/γ agonists, have attracted global attention due to unique lipid and glycemic modifying actions [9, 33, 36]. Many Glitazars such as Muraglitazar, Ragaglitazar, Tesaglitazar, Naveglitazar, Farglitazar, Aleglitazar were developed but failed during preclinical stage or the clinical development stage due to lack of efficacy or safety issues [9, 36–38]. Clinical development of Muraglitazar was discontinued due to cardiovascular AEs such as myocardial infarction, stroke, heart failure [9]. Clinical development of Aleglitazar was discontinued due to AEs such as heart failure, gastrointestinal bleeding, and renal dysfunction [9, 38].

Saroglitazar is the first and only dual PPAR α/γ agonist (Glitazars) to be approved as well as prescribed in clinical practice, anywhere in the world [9, 33]. Following marketing authorization in India (2013), Saroglitazar was also granted marketing authorization in Mexico (2017). This is the first review to summarize the effects of Saroglitazar in patients with diabetic dyslipidemia in real world clinical studies conducted after its marketing approval in India in 2013. In this review, we reviewed 18 articles including 5 published manuscripts and 13 abstracts. In total, 5824 patients with diabetic dyslipidemia received Saroglitazar 4 mg for durations ranging from 12 weeks to 58 weeks in the selected studies.

Kaul et al. [21] was the first study to examine the effects of Saroglitazar on non-HDL-C as the primary endpoint and sd-LDL-C as a secondary endpoint in 104 patients with diabetic dyslipidemia. The authors observed significant decrease in non-HDL-C (baseline: 142.3 ± 59.3 mg/dL to week-24: 109.9 ± 45.5 mg/dL), sd-LDL-C (baseline: 32.5 ± 11.3 mg/dL to week-24: 25.9 ± 11.8 mg/dL), HbA1c (baseline: 8.1 ± 1.7 (%) to week-24: 6.9 ± 0.7 (%)), and significant increase in HDL-C (baseline: 37.3 ± 18.4 mg/dL to week-24: 43.4 ± 15.6 mg/dL) in the per-protocol population [21]. Shetty et al. [13] was the largest observational study of Saroglitazar in 2804 patients with diabetic dyslipidemia. The authors observed significant decrease in TG (baseline: 312.3 ± 122.7 mg/dL to week-12: 188.7 ± 61.4 mg/dL), non-HDL-C (baseline: 201.8 ± 64.1 mg/dL to week-12: 149.4 ± 41.0 mg/dL), HbA1c (baseline: 8.3 ± 1.3 (%) to week-12: 7.4 ± 0.9 (%)), and significant increase in HDL-C (baseline: 38.8 ± 8.7 mg/dL to week-12: 41.0 ± 7.1 mg/dL) [13].

Joshi et al. [15] conducted a postmarketing surveillance study of Saroglitazar in 18 T2DM patients with severe hypertriglyceridemia (baseline TG ≥ 1000 mg/dL). The authors observed significant decrease in TG (baseline: 1265.9 ± 394.3 mg/dL to week-12: 402.0 ± 221.8 mg/dL), non-HDL-C (baseline: 320.8 ± 172.8 mg/dL to week-12: 176.4 ± 62.9 mg/dL), and HbA1c (baseline: 8.9 ± 1.7 (%) to week-12: 7.8 ± 0.9 (%)) [15]. Chatterjee et al. [30] conducted a 58 weeks observation study of Saroglitazar in 158 patients with diabetic dyslipidemia (baseline TG ≥ 150 mg/dL). The authors found significant reduction in TG (baseline: 319.9 ± 178.8 mg/dL to week-58: 174.0 ± 113.6 mg/dL), non-HDL-C (baseline: 140.1 ± 55.4 mg/dL to week-58: 104.5 ± 49.7 mg/dL), and HbA1c (baseline: 7.9 ± 1.5 (%) to week-58: 7.3 ± 1.4 (%)) [30].

Three studies included NAFLD patients with diabetic dyslipidemia [17, 18, 22]. Joshi et al. [18] conducted a single centre, single arm, prospective, open label study in 221 patients with diabetic dyslipidemia also diagnosed for NAFLD by transient elastography (FibroScan™). The authors found that Saroglitazar significantly reduced TG (baseline: 321.0 mg/dL to week-24: 129.0 mg/dL) and ALT (baseline: 89.0 IU/L to week-24: 21.0 IU/L) and improved fatty liver (evaluated by transient elastography (FibroScan™)) in 39% (86/221) patients [18]. Similarly, Saboo et al. [17] observed that Saroglitazar significantly decreased TG (baseline: 259.3 ± 37.9 mg/dL to week-24: 151.5 ± 53.6 mg/dL) and ALT (baseline: 64.1 ± 6.2 IU/L to week-24: 28.7 ± 3.2 IU/L) in 31 NAFLD patients with diabetic dyslipidemia. Goyal et al. [22] also found that Saroglitazar significantly decreased TG (baseline: 334.7 ± 74.0 mg/dL to week-24: 158.5 ± 46.0 mg/dL) and ALT (baseline: 98.0 ± 32.0 IU/L to week-24: 34.0 ± 14.0 IU/L) in 84 patients with diabetic dyslipidemia (78.5% of these 84 patients were diagnosed for NAFLD by transient elastography (FibroScan™)).

One excluded study, STOP-D, was a prospective, single centre, single arm study in 40 patients with pre-diabetes (baseline HbA1c: 5.7-6.4%) and dyslipidemia (TG > 150 mg/dL, total cholesterol > 200 mg/dL, LDL-C > 130 mg/dL and HDL-C < 40 mg/dL) [39]. The authors found that Saroglitazar significantly reduced TG (baseline: 348.0 ± 86.9 mg/dL to week-24: 216.4 ± 72.3 mg/dL) and HbA1c (baseline: 6.3 ± 0.2% to week-24: 5.5 ± 0.3%) [39].

In Phase-3 clinical trials, most frequently reported AEs (≥ 2% of patients) with Saroglitazar 4 mg use were asthenia, gastritis, dizziness, tremors in the PRESS V study and were gastritis and pain in the PRESS VI study [11, 12]. In real world clinical studies, Kaul et al. [21] reported hypoglycemia after first dosing to be Saroglitazar related AE.

In all included studies, Saroglitazar favourably modulates lipid and glycemic parameters without significant AEs in patients with diabetic dyslipidemia [13–30]. Saroglitazar 4 mg effectively reduced lipid parameters—TG, TC, LDL-C and non-HDL-C, glycemic parameter—HbA1c, and effectively increased lipid parameter—HDL-C in patients with diabetic dyslipidemia [13–30]. Saroglitazar has a potential to address the residual cardiovascular risk associated with high non-HDL-C, high TG, and low HDL-C in patients with diabetic dyslipidemia [7, 8]. The Emerging Risk Factors Collaboration, in a study of more than 300,000 people without initial vascular disease from 68 long-term prospective studies, found that non HDL-C was the strong predictor for coronary heart disease (CHD) (50% increased risk) and ischemic stroke (12% increased risk) [2]. Toth et al. conducted an observational administrative claims analyses of statin-treated patients aged ≥ 45 years with high residual cardiovascular risk [40]. In a multivariate analysis, patients with TG (200-499 mg/dL) (n = 13,411), compared to patients with TG (< 150 mg/dL) and HDL-C (> 40 mg/dL) (n = 32,506), was associated with 35% increased risk of composite major cardiovascular events, 35% increased risk of nonfatal myocardial infarction, and 27% increased risk of nonfatal stroke [40]. The Emerging Risk Factors Collaboration also found that HDL-C was associated with 22% decreased risk of CHD [2].

Saroglitazar was also found effective in lowering ALT [17, 18, 22] and improving fatty liver (evaluated by sonographic (FibroScan™) investigation) in NAFLD patients with diabetic dyslipidemia [18]. These results are also supported by a preclinical study conducted by Jain et al. [41]. In experimental non-alcoholic steatohepatitis (NASH) model (animal model of mice with choline-deficient high-fat diet-induced NASH), the authors found that Saroglitazar reduced ALT, hepatic steatosis, inflammation, ballooning, and prevented fibrosis development [41]. At present, there are no medications approved for NAFLD/NASH [42]. Saroglitazar could become a promising treatment option for NAFLD/NASH [17, 18, 22]. However, the efficacy and safety of Saroglitazar in patients with NAFLD/NASH must be examined in RCTs. At present, several RCTs are going on in the USA (ClinicalTrials.gov Identifier: NCT03061721) and India (CTRI/2015/10/006236) to study the effects of Saroglitazar in patients with NAFLD/NASH.

There are strength and weakness of all selected studies in this review. There is a possibility of under-reporting of AEs due to lost to follow-up in studies based on real-world clinical settings [21, 30]. Moreover, there are 13 abstracts out of 18 selected studies and we cannot critically review these studies due to limited information available in the abstracts. However, these abstracts were presented at the top global annual scientific congress such as the scientific sessions of the American Diabetes Association, the annual scientific congress of the American Association of Clinical Endocrinologists, and the Annual Conference of Asian Pacific Association for the Study of the Liver. Moreover, all the selected studies included patients from the real-world clinical settings that certainly improves the generalizability of the study results of all selected studies [21, 30]. Additionally, the study results from Chatterjee et al.dyslipidemia [30] supports long-term effectiveness and safety of 58 weeks for Saroglitazar in patients with diabetic dyslipidemia.

## Conclusion

In summary, Saroglitazar effectively improved lipid and glycemic parameters without significant AEs in patients with diabetic dyslipidemia in real-world clinical studies of up to 58 weeks duration.

## Data Availability

Our study is a review article. All data reviewed or analysed in this review article were extracted from 18 selected studies (5 published manuscripts and 13 abstracts) available in the public domain as mentioned in ‘References’ section of the manuscript.

## Abbreviations

CVDs: cardiovascular diseases
T2DM: type 2 diabetes mellitus
TG: triglyceride
sd-LDL-C: small-dense low-density lipoprotein cholesterol
LDL-C: low-density lipoprotein cholesterol
HDL-C: high-density lipoprotein cholesterol
non-HDL-C: non high-density lipoprotein cholesterol
PPAR: peroxisome proliferator activated receptor
NAFLD: non-alcoholic fatty liver disease
NCBI: National Center for Biotechnology Information
TC: total cholesterol
RCTs: randomized controlled trials
HbA1c: glycosylated hemoglobin
ALT: alanine aminotransferase
AEs: adverse events
NASH: non-alcoholic steatohepatitis
CHD: coronary heart disease
